# Cohesin-Mediated Chromatin Interactions and Autoimmunity

**DOI:** 10.3389/fimmu.2022.840002

**Published:** 2022-02-10

**Authors:** Venkataragavan Chandrasekaran, Nina Oparina, Maria-Jose Garcia-Bonete, Caroline Wasén, Malin C. Erlandsson, Eric Malmhäll-Bah, Karin M. E. Andersson, Maja Jensen, Sofia T. Silfverswärd, Gergely Katona, Maria I. Bokarewa

**Affiliations:** ^1^Department of Rheumatology and Inflammation Research, Institute of Medicine, University of Gothenburg, Gothenburg, Sweden; ^2^Rheumatology Clinic, Sahlgrenska University Hospital, Gothenburg, Sweden; ^3^Department of Medical Biochemistry and Cell Biology, Institute of Biomedicine, University of Gothenburg, Gothenburg, Sweden; ^4^Ann Romney Center for Neurologic Diseases, Harvard Medical School, Brigham and Women’s Hospital, Boston, MA, United States; ^5^Department of Biology and Biological Engineering, Chalmers University of Technology, Gothenburg, Sweden; ^6^Department of Chemistry and Molecular Biology, Faculty of Science, University of Gothenburg, Gothenburg, Sweden

**Keywords:** cohesin, CCCTC-binding factor, autoimmunity, genome organization, immune signaling

## Abstract

Proper physiological functioning of any cell type requires ordered chromatin organization. In this context, cohesin complex performs important functions preventing premature separation of sister chromatids after DNA replication. In partnership with CCCTC-binding factor, it ensures insulator activity to organize enhancers and promoters within regulatory chromatin. Homozygous mutations and dysfunction of individual cohesin proteins are embryonically lethal in humans and mice, which limits *in vivo* research work to embryonic stem cells and progenitors. Conditional alleles of cohesin complex proteins have been generated to investigate their functional roles in greater detail at later developmental stages. Thus, genome regulation enabled by action of cohesin proteins is potentially crucial in lineage cell development, including immune homeostasis. In this review, we provide current knowledge on the role of cohesin complex in leukocyte maturation and adaptive immunity. Conditional knockout and shRNA-mediated inhibition of individual cohesin proteins in mice demonstrated their importance in haematopoiesis, adipogenesis and inflammation. Notably, these effects occur rather through changes in transcriptional gene regulation than through expected cell cycle defects. This positions cohesin at the crossroad of immune pathways including NF-kB, IL-6, and IFNγ signaling. Cohesin proteins emerged as vital regulators at early developmental stages of thymocytes and B cells and after antigen challenge. Human genome-wide association studies are remarkably concordant with these findings and present associations between cohesin and rheumatoid arthritis, multiple sclerosis and HLA-B27 related chronic inflammatory conditions. Furthermore, bioinformatic prediction based on protein-protein interactions reveal a tight connection between the cohesin complex and immune relevant processes supporting the notion that cohesin will unearth new clues in regulation of autoimmunity.

## Introduction

Genomic content inside the nucleus exists at different levels of organization. A few of these levels are contact domains or topologically associating domains (1), chromosomal compartments (2), chromosomal loops (3, 4), phase-separation (5), and nucleosomes. Depending on the activity within the nucleus, any or all the chromosomal organizational levels can coexist together. In the past decade, several studies of chromatin interaction and their effects on gene transcription, and vice versa, have invariably found that chromatin organisation is important for cell division, maturation, and normal physiological function of any cell.

The cohesin complex controls gene expression programs through chromatin looping. Cohesin was initially thought to perform its main function only in the context of sister chromatid cohesion and chromosome segregation. However, the rise of chromatin conformation capture (3C) techniques directed the attention to hitherto unidentified functional properties of cohesin including its highly dynamic binding to DNA maintained by proteins CDC5A and WAPL (6), and its early loading on chromatin prior to the onset of S phase of the cell cycle through the NIPBL-MAU2 loader complex (7). These observations emphasized unrecognized roles of cohesin in regulation of transcription, and 3D genome organization. CTCF is an architectural protein, which in partnership with the cohesin complex organizes the three-dimensional structure of the genome in most cells. CTCF binding sites are distributed throughout the genome and their occupancy dictate efficient chromatin organisation to regulate gene transcription in the cells of both adaptive and innate immunity. In addition to cohesin’s role in establishing cohesion, it also performs other functions such as facilitation of enhancer-promoter contacts, establishment of insulator domains through loop extrusion, three-dimensional organization of the genome, aiding DNA replication and DNA repair, and encouraging long-range interactions between different gene loci.

Non-homeostatic microenvironments are characteristic features of autoimmune conditions such as rheumatoid arthritis (RA), type 1 diabetes (T1D), and systemic lupus erythematosus (SLE). In response, immune cells in different niches of the body acquire aberrant gene regulation caused by alterations in proper genome networks. The advanced technique of chromosome conformation capture (3C) helps in picking apart the complex networks formed within different genomic loci. It is common practice in the GWAS studies to associate disease SNPs to the nearest known gene. However, capture Hi-C analyses questioned this practice and demonstrated that cohesin complex mediated numerous long-range interactions of the SNP-containing chromatin regions with distal promoters and enhancers (8). Integrating proteomic and transcriptomic data within this framework could help in strengthening causality between gene polymorphisms and phenotypic characteristics.

This review explores recent emerging evidence on the role of cohesin in enabling long-range chromatin interactions and mediating multiple regulatory networks in cells of adaptive and innate immunity with potential implications in autoimmunity.

## Structure and Function of Cohesin Complex

**Figure 1:**
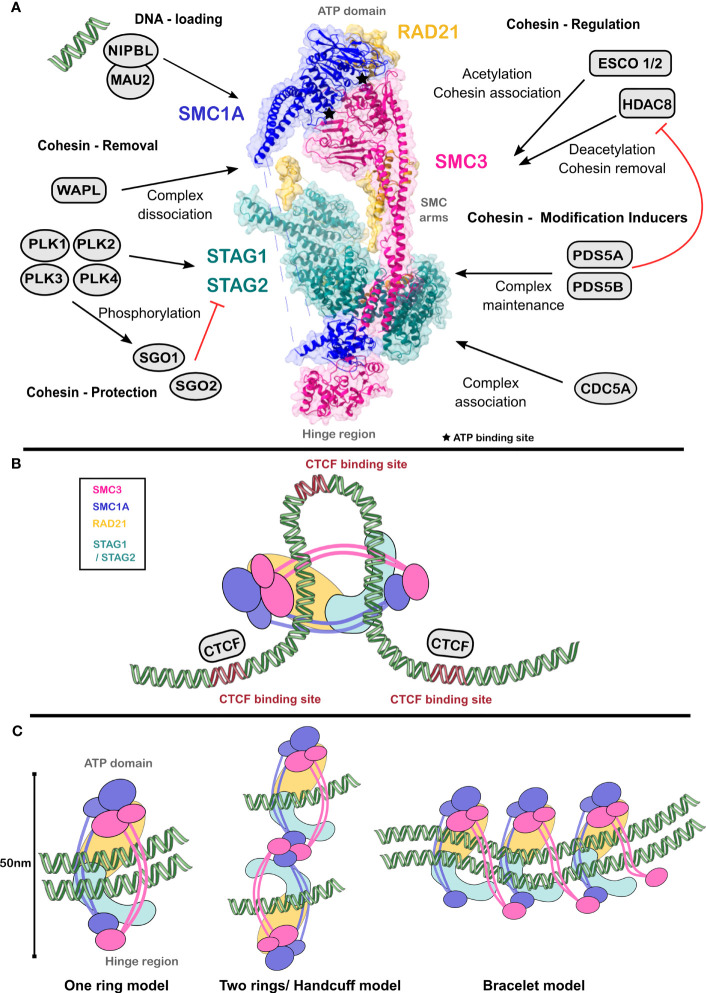
Cohesin complex enables chromatin architecture. **(A)** SMC1 and SMC3 heterodimer forms the ‘hinge’ region and the ATPase domains at either ends. RAD21 and STAG1/STAG2 complete the cohesin complex. Several proteins dynamically bind to the cohesin complex and regulate its function. Structure of cohesin complex was obtained from PDB ID 6wg3 (10). **(B)** Cognate binding sites of CTCF (in purple) constrain cohesin translocation, thus forming chromosomal loops. **(C)** Mechanistic models of cohesin action dynamically exist within a cell to enable cohesin-mediated chromatin organization. Figures were created using Chimera, Inkscape and the Servier Medical Art.

The cohesin complex is a member of the larger family of SMC complexes that include condensin I and condensin II in humans (9). Orthologs of these complexes may be found in yeast, and in *C. elegans*. SMC3, SMC1s, RAD21 and STAG1/2 form the core cohesin complex (**Figure 1A**), conserved across different species ranging from yeast to vertebrates (11). Several protein groups are important for cohesin to perform its canonical functions within the nucleus. These groups include the core cohesin complex consisting of SMC1(A,B), SMC3, RAD21 and STAGs; the loading proteins NIPBL, MAU2; the cohesin regulating proteins ESCO1, ESCO2, HDAC8; the proteins inducing cohesin modifications PDS5A, PDS5B, CDC5A; and finally, the removal proteins WAPL, PLKs, SGO1, SGO2.

SMC1(A or B) and SMC3 form a heterodimer by interacting through the hinge region at one end and the ATP head domain, which has ATPase activity, at the other end (**Figure 1A**). These two regions are separated by an α-helix coiled-coil fibre (12), which provides a V-like structure to the complex. By the binding of PDS5 and ATP-mediated association of RAD21, the complex forms a triangular ring allowing DNA entry, entrapment, and exit. CTCF binding sites oriented in a reverse-forward direction constrain cohesin translocation through the genome (13), enabling transient DNA looping (**Figure 1B**).

Structural studies on the cohesin complex with cryo-electron microscopy (10) and site-specific mutations allowed proposal of different models of its mechanism of action (**Figure 1C**). The early *one ring model* suggests that a single cohesin ring establishes the attachment of both sister chromatids after replication. This model does not explain the dynamic nature of cohesin activities, such as its loading on sister chromatids and unloading during different stages of the cell cycle, as well as maintenance of sister chromatid cohesion during DNA replication. The *handcuff model* argues for interaction between two or more cohesin complexes through two RAD21 molecules each binding to SMC1 and SMC3 (11). Here, each complex entraps only one sister chromatid. Finally, the *bracelet* model, proposes the formation of oligomerized bracelets between cohesin complexes where RAD21 contacts both SMC1 and SMC3 (14). Although the *bracelet model* is generally accepted by the scientific community, the proposed models are not mutually exclusive. Dynamic switching between the states mediated by events such as phosphorylation, oligomerization, and protein-protein interactions within the cohesin complex and with other nuclear proteins opens up the possibility for a condition-dependent use of all the proposed models.

## Canonical Function of Cohesin Proteins Within the Nucleus

Cohesin is loaded on nucleosome complexes through the sequential concerted action of the above proteins (**Figure 1A**). Primarily, the NIPBL-MAU2 loader complex in humans, engages cohesin with the chromosomes and triggers the ATPase head domains of SMC1 and SMC3 (7, 15). DNA enters and exits the cohesin complex (16) by interacting with cohesin complex subunits NIPBL (10) and PDS5 (17). A 72-base DNA substrate comprised of A-T rich sequences was found to stabilize this complex. ATP-binding at the head domains is sufficient to establish DNA interactions. However, a complete entrapment is achieved by ATP hydrolysis and engagement of the DNA with lysines of the SMC1-SMC3 heterodimer. Electron cryo-microscopy observations (10) showed that the yeast ortholog of SMC1-SMC3, Psm1-Psm3, binds to the yeast STAG1/2 ortholog Psc3. This association potentially maintains orientation of the DNA in an appropriate conformation to establish contacts between the DNA, SMC1/SMC3, NIPBL, and RAD21 (16).

Once the DNA is bound, the cohesin complex needs to be stably maintained. PDS5 mediates acetylation of the SMC3 head domains by ESCO1/2 (17), thus simultaneously preventing HDAC8 dependent deacetylation. PDS5 binds to SMC3 in proximity to STAG1, which maintains entrapped DNA molecules during S phase. Additionally, PDS5 causes recruitment of the protein WAPL to enable cohesin release from chromosomal arms during the prophase of mitosis (17). The WAPL-mediated removal of cohesin from the centromere is protected by CDC5A in competition with PDS5 (18). Alternatively, cohesin is maintained on chromatin through the action of SGO1 and SGO2 proteins that function at the pericentromeric region during mammalian mitosis and inhibit WAPL to prevent premature cohesin release (19). Successive phosphorylation events of the Spindle Assembly Checkpoint cause the weakening of CDC5A-PDS5 interaction, freeing WAPL. Simultaneously, phosphorylation of RAD21 by PLK1 activates the protein separase/ESPL1 to promote entry into anaphase.

## 3D Genome Organization and Regulation of Gene Transcription

Apart from its known role in the cell cycle, recent evidence has postulated non-canonical roles for cohesin in the nucleus. This notion has primarily arisen since it has been observed repeatedly that levels of cohesin within the nucleus exceed the required amount for cell cycle progression (20). Additionally, severe effects not always connected to the cell cycle, were observed in both humans and mice when one or more of the cohesin complex proteins were disturbed. Such discordant scenarios puzzled researchers and nudged them towards exploring the role of cohesin in a wider context. The insulator protein CCCTC-binding factor (CTCF) provided a clue into this puzzle.

CTCF is a zinc finger protein and the partner of cohesin complex in insulating/protecting chromatin elements. CTCF is essential for the insulation activity of cohesin on chromatin (13). Cohesin complex progressively moves through the genome using both passive diffusion and RNA polymerase activity. This causes chromatin loops to form until they encounter chromatin-bound CTCF (13). This phenomenon is generally termed as the ‘loop extrusion’ activity of cohesin. The energy for loop extrusion is obtained through intrinsic ATPase activity, while it has been shown that ATP is dispensable for further maintenance of chromatin loops once established (21). The reverse-forward orientation of CTCF binding sites on the genome organizes chromatin three-dimensionally, forming ‘Topologically Associating Domains’ (22) (**Figure 1B**) Loop extrusion performed by dynamic travel of cohesin on chromatin is vital for proper gene expression. It reduces contact between non-specific enhancers and enhancer-blocking genes and encourages specific promoter-enhancer effects for recruitment of transcription factors to distinct chromosomal domains and initiation of gene expression. Single cell Hi-C data from haploid mouse embryonic stem cells showed that previously identified cohesin-CTCF loops (2) interact with boundaries of the active chromatin recognized by its open conformation, presence of gene-activating histone modifications and active gene expression (23).

Hi-C, FRAP, and ChIP experiments demonstrated that the duration of cohesin engagement of chromatin is far shorter than CTCF’s chromatin engagement (1 and 20 minutes, respectively) (24). Hence, each of these proteins had to be performing overlapping and non-overlapping tasks within the highly condensed, yet functionally ordered, nuclear environment. On an overall scale in a large cell population, both cohesin and CTCF are integral to the formation of chromatin loops. However, their exact roles in every specific moment of this process remain to be defined. ChIP-Seq analysis reveals that 75% of the total fraction bound by cohesin is involved in loop formation while the CTCF-bound fraction is only 37% (24). Additionally, heights of CTCF peaks were generally low at antigen receptor loci during maturation of B and T cells, indicating rapid formation and dissolution of chromatin elements (25). CTCF is expected to be majorly involved in the initial process of demarcating insulator DNA elements and constantly shuffles through the genome to bind cognate sites elsewhere (24). CTCF’s role is exemplified through the canonical observations done on the *Igf/H19* locus (26). Here, maternal allele-specific binding of CTCF is associated with increased demethylation and gene expression and reorganization of the local chromatin domains, while the corresponding paternal allele is associated with low gene expression, absence of CTCF binding and increased DNA methylation. Thus, CTCF and cohesin have co-essential roles in the context of loop extrusion and in the larger context of effecting long-range chromatin interactions that translate into gene expression. It is also important to note that CTCF has several individual functions independent of its interaction with cohesin, such as DNA binding, RNA Polymerase II elongation, and involvement in splicing (27), which are outside the scope of this review.

To identify which parts of the cohesin complex interact with CTCF, ChIP-chip experiments were performed (28, 29). Several co-localization sites for STAG1 and CTCF were found. Abolishing those CTCF-STAG1 colocalization sites by mutation blocked cohesin binding (28). In another study employing CTCF mutant constructs, STAG2 was found to interact with the C-terminal tail of CTCF (29). The differences in CTCF binding to STAG1 and STAG2 could explain why proteins with high sequence homology perform different biological roles (30).

## Expression of Cohesin Proteins in Immune Cells

Cohesin deletion affects development and the immune system drastically. Analysis of transcriptomic data using GTEx tissue expression profiling, BLUEPRINT EpiVar, and Immune Cell Atlas demonstrated that immune cell subsets of lymphoid and myeloid lineage showed consistently high expression of the core cohesin complex genes i.e., *SMC1A, SMC3, RAD21, STAG1/STAG2* across the examined sources. Essential cohesin interacting partners *PDS5A/5B, WAPL* and *NIPBL* were also highly expressed. Immune-relevant tissues obtained from GTEx uniformly revealed high expression of the cohesin complex proteins. Canonical and non-canonical functions exhibited by the core cohesin complex, or its associated proteins are occasionally contingent on the protein isoform and/or its dosage. For example, *SMC1A* level was high and widely expressed across immune cells, while *SMC1B* was low or undetectable, and haploinsufficient *SMC3* resulted in germinal center (GC) hyperplasia while its complete deletion abolished GC formation (31). *STAG2* is expressed higher in most samples in comparison to *STAG1*. STAG2 and STAG1 share 70% sequence homology, with STAG2 being dominant in somatic (29) and haematopoietic stem cells.

## Monogenic Disorders Associated With Cohesin Dysfunction

Clinical consequences of insufficient cohesin function because of mutations in the cohesin subunits and/or dysregulated sister chromatid cohesion are the underlying causes of disorders called ‘cohesinopathies’. The most famous among them is the Cornelia de Lange Syndrome (CdLS), which is an autosomal dominant (NIPBL, SMC3, RAD21) and X-linked (SMC1 and HDAC8) disease with almost complete penetrance (32, 33). It has high intra-familial phenotype variability being frequently characterized by abnormal facial and intellectual development, gastrointestinal and heart disorders, and limb malformation (33). *NIPBL* is the major and densely mutated gene found in about 75% of CdLS cases. Even a 15% reduction in the levels of *Nipbl* mRNA causes a radical increase in susceptibility to CdLS-like phenotype in mice (34). Mutations in other cohesin complex subunits such as SMC3 (35) and HDAC8 (36) have also been discovered. A minor percentage of the cases in CdLS were linked to copy number variants in *RAD21*, associated with the non-classic CdLS phenotype variants (37). Most of the *RAD21* variants were truncated or contained missense mutations, which commonly affected production of RAD21 protein. Similarly, a truncated ESCO2 protein is observed in patients with Juberg-Hayward syndrome (38) and Roberts syndrome (39). It causes a multitude of musculoskeletal defects in the face and in limbs with overlapping and divergent phenotypes observed in each. Inherited cohesion defects and symptoms similarity demonstrated close functional association between the underlying mutated genes. However, reports on deviation in haematopoiesis and immune system development in patients with CdLS do not exist today.

Since cohesin is closely linked to the normal progression of mitosis, one would expect aneuploidy or cell cycle dysregulation to be among the most common processes affected due to cohesin complex alterations. Interestingly, this is not the case (40, 41). As studies on cohesinopathies continue to emerge, it becomes obvious that part of these pathologies is associated with impaired gene expression regulation (42, 43). With the exception for CdLS, no monogenic diseases were found for other genes of the cohesin complex. This could partly be explained by importance of these genes in embryogenesis and organ development, where mutations could often be lethal. A diversity of roles played by individual protein subunits within SMC complexes could present a different explanation to the low number of pathologies associated with these genes. Short life span and numerous organ and skeletal malformations combined with intellectual disability of survivors defend low priority for collection of immunological data from such patients.

## Clinical Traits in Proximity of the Cohesin Protein Genes

An important cluster of information arises from the GWAS single nucleotide polymorphism (SNP) studies summarized using ClinGen and PhenGenI databases along with the EBI GWAS catalogue studies (**Table 1**). For variants located exactly within the gene(s), significant immunologically relevant associations were found for CTCF (eosinophil WBC count), MAU2 (basophil WBC count), PDS5B (asthma), SGOs (multiple sclerosis, IgG glycosylation, macrophage driven inflammation), SMC1B (RA), STAG1 (RA, lymphocyte cell count, C-reactive protein levels). An example of cohesin complex importance in a canonical autoimmune disorder has been recently presented in a large multiple ancestral GWAS analysis (44). The study involved European and East Asian cohorts and identified *SMC1B* as one of the novel GWAS loci independently associated with susceptibility to RA (44). Clinical implication of SMC1B in immunological processes in RA awaits further research. Since gene-specific regulatory associations could cover much larger genomic distances, we additionally checked the traits in 100kb vicinity of these loci (**Table 1**). Indeed, this selection of immunologically relevant traits was enriched in other SNPs associated with lymphocyte counts, asthma and lung function, C-reactive protein levels, RA and, included a set of chronic inflammatory diseases traditionally associated with the genetic HLA-B27 background including ankylosing spondylitis, Crohn’s disease, psoriasis, primary sclerosing cholangitis, and ulcerative colitis. This highlights the importance of further research connecting the cohesin complex genes and immune system phenotypes.

**Table 1:** GWAS based SNPs in the cohesin complex proteins in humans.

Gene	Chromosome	Variant ID	Trait/Phenotype	p-value	Reference PMID
*CDCA5*	11	rs11227126	**Crohn’s disease**	2.00E-06	32581322
*CTCF*	16	rs113028056	Eosinophil % WBC	1.00E-13	32888494
		rs80190634	Hemoglobin concentration	8.00E-10	32888493
		rs117556162	Eosinophil counts	1.00E-15	30595370
			Lymphocyte Counts	9.00E-24	32888494
				1.00E-26	32888493
				3.00E-27	
		rs11867039	Lymphocyte Counts	7.00E-11	32888494
			Monocyte count	7.00E-11	
		rs7196853	Lung function	6.00E-26	30595370
		rs77971272		2.00E-10	
*MAU2*	19	rs2285627	Basophil WBC count	1.00E-09	27863252
		rs58542926	C-reactive protein levels	3.00E-18	33462484
				1.00E-25	31900758
		rs10401969		2.00E-32	27286809
*PDS5B*	13	rs188887209	**Asthma**	4.00E-06	27611488
*PLK1*	16	rs55869023	Eosinophil counts	7.00E-09	32888494
		rs66815895		1.00E-09	30595370
*SGO1*	3	rs74696548	**Multiple Sclerosis**	3.00E-06	33296963
		rs6787231	IgG glycosylation	5.00E-06	23382691
		rs2623079	WBC count	1.00E-08	30595370
*SMC1B*	22	rs35156883	**Rheumatoid arthritis**	2.00E-08	33310728
		rs1569414	HLA-B27 associated diseases*	6.00E-10	26974007
*SMC3*	10	rs10787268	Lung function	4.00E-12	30595370
*STAG1*	3	rs9826828	**Rheumatoid arthritis**	9.00E-08	30423114
				9.00E-10	24390342
		rs7621025	C-reactive protein levels	1.00E-09	27286809
		rs9835571	Lymphocyte counts	3.00E-27	32888493
		rs9834250		9.00E-17	32888494
			Monocyte count		

*Ankylosing spondylitis, Crohn’s disease, psoriasis, primary sclerosing cholangitis, ulcerative colitis.

**Diseases that have SNP associations.**

SNP is found within 100kb of the gene

## Somatic Mutations in Cohesin Proteins and Hematological Malignancies

It has been recently reported that somatic mutations in the cohesin complex genes could be associated with myeloid (30, 45, 46), and lymphoblastic leukemias (47, 48) suggesting their importance at the early stages of leukopoiesis. For example, analysis of cohesin complex mutations in large cohorts of patients suffering myelodysplastic syndrome (MDS) and acute myeloid leukemia (AML) (40) showed that several members of the cohesin complex were mutated in bone marrow and blood cells, which resulted in haploinsufficient levels of those cohesin proteins. *STAG2* was mutated in 18-50% of the cases, while *RAD21* in 30-47% and *SMC3* in 18-30%. The co-occurring mutations included genes encoding basic transcriptional regulators *RUNX1* and *BCOR*, and chromatin modifiers *ASXL1, SRSF2* and *TET2*. The presence of mutations was associated with the overall poor survival, specifically the MDS patients carrying STAG2 mutations had short survival time.

Cohesin loss in early hematopoietic progenitors affected the architecture of chromatin surrounding important hematopoietic genes. Haplo-insufficiency of RAD21, STAG1/STAG2, or SMC1-SMC3 caused increased self-renewal of the hematopoietic progenitor cells, at the expense of differentiation (41). The aberrant chromatin architecture led to unwanted gene expression required for the proliferative phenotype. Analysis of RNA-Seq of AML patients in TCGA found a significant down-regulation of IFNα, IFNγ and TNFα signaling pathways, which resulted in inability to control multiple immunologically relevant processes (49). Disruption of RAD21/cohesion integrity in hematopoietic stem and progenitor cells (HSPCs) resulted in remarkable deviations in NF-kB signaling required for inflammation and differentiation (50). A nonsense mutation in *RAD21* leads to a dysfunctional protein and affects expression of *Runx1*, a master hematopoietic gene regulator (37). Similarly, MDS and AML display a coexistence of *STAG2* and *RUNX1* mutations (40). Chromatin organisation studies confirmed that a combined deletion of these proteins impairs enhancer-promoter loops and selectively affects transcriptional activity (51).

## Cohesin-CTCF Dependent Genome Organization in Autoimmune Diseases

Understanding the genome organization in the loci of interest is often the first step in identifying the mechanisms and the underlying causes for aberrant transcription. Supporting this, altered distribution of cohesin/CTCF complexes in CD4^+^ T cells has been described in association with different autoimmune phenotypes and in essential immunological processes.

CD4^+^ T cells isolated from patients with SLE, RA and Sjögren’s syndrome had differential expression of PP2A B55β, a protein involved in cytokine withdrawal-induced cell death (52). DNA methylation levels in the PP2A B55β gene were high both in RA and in SLE, which was associated with reduced CTCF binding. Analysis of coordinated gene expression pattern in SLE patients and healthy controls identified the disease activity dependent deregulation in IFN pathway, indicating genome structure and organisation to make important contribution in gene expression and the disease activity (53). Inquiring into regulation of RA relevant genes, *FOXO1* and *MYC*, studies on TCR activated CD4^+^ T cells showed that the compartments containing open, expression-active or closed, expression-inactive chromatin and TADs remained largely stable (54). However, dynamic changes occurred within the organizational structure and a transient enrichment of CTCF binding sites in regions of open chromatin was observed.

CD4^+^ T cells of patients with active juvenile idiopathic arthritis (JIA) showed different transcriptional profiles compared to patients in remission and healthy controls, which were associated with differences in chromatin organization, and potentially, in occupancy of CTCF binding sites (55). CD4^+^ T cells of patients with systemic sclerosis were different from healthy in DNA methylation around HLA gene clusters and genes enriched in inflammatory pathways. Furthermore, CTCF binding was enriched in both hyper- and hypomethylated CpG positions in CD4^+^ cells of scleroderma patients (56). In patients with acute Graft-versus-Host Disease, p53 was identified as an important regulator of the IL-2 induced proliferative response of CD4^+^ T cells. Expression of p53 was regulated by CTCF binding to its promoter region, which recruited the histone acetylase p300 to acetylate H3K9 and H3K14. Low CTCF expression caused an insufficient proliferative response to IL-2 and skewed T cells towards the Th17 phenotype (57).

GWAS studies have been widely used to identify risk genes for autoimmune diseases. SNPs associated with MS were mapped in a 600kb region of chromosome 16, which contained the intron to the gene *EVI5* and the regulatory domain of *GFI1* (58). The linkage disequilibrium patterns in the region and logistic regression analysis of the associations suggested presence of novel MS disease loci. Expression QTLs, histone modifications and CTCF binding data in the region revealed the identified SNPs potentially prevented interaction between the *EVI5* intron and the *GFI1* promoter through three CTCF binding sites (58). The independent genetic signal affected the CIITA-CLEC16A-SOCS1 gene complex (59) and proposed novel research efforts towards studies on functional epigenetic regulation in pathogenesis of MS.

Autoimmune type 1 diabetes (T1D) in both humans and non-obese diabetic (NOD) mice is characterized by leukocyte infiltration of pancreatic islets, ultimately leading to the destruction of insulin-producing islet beta cells. Genetic analysis of the NOD mice identified 18 megabase pair domains, called “insulin-dependent diabetes” (*Idd*) regions, controlling diabetes susceptibility in NOD mice (60). Comparison of genomic architecture in thymocytes by optical mapping of chromosomal contact domains showed misfolding of *Idd* regions in NOD mice and in immune cells isolated from pancreas tissue of T1D patients (60). Comparison of chromatin accessibility of thymic T cells revealed pre-patterning of the genome at select *Idd* regions that occurred prior to the disease onset. In NOD mice, this was associated with differential expression pattern of the genes located in the *Idd*-regions ontologically annotated to the “regulation of leukocyte-mediated cytotoxicity” and “signaling by interleukins” pathways. High-throughput chromosome conformation capture (Hi-C) analyses using cohesin subunit SMC1 clearly showed distinct cis-looping at bound areas of hyperconnected regions of *Bcl11b, Runx1* and *Ets1 and Cd8* regions important for development of T cells.

## Effect of Cohesin Manipulation in Mice

The studies above implied 3D genome configuration as a molecular contributor to autoimmunity. However, scientific literature exploring cohesin’s importance to hematopoiesis and immune system development is limited by the lack of available human data linked to mutations in cohesin proteins. Hypothetically, this occurs because of devastating consequences of the mutations on embryonic development that, in most cases, cause lethality. Thus, we chose to infer cohesin’s importance in humans by analysing the literature on mouse models where cohesin proteins were manipulated.

In mouse studies, embryonic or neonatal lethality are described for CDC5A, HDAC8, CTCF, ESCO2, MAU2, NIBPL, PDS5A, PDS5B, PLK1, PLK2, PLK4, RAD21, SCO1, SMC3, STAG1, STAG2 and WAPL (**Table 2**). Results in different studies could vary showing complete or incomplete penetrance for a particular genetic defect. Several important findings from gene modification approaches in cell lines and mouse stem cell studies reveal importance of cohesin and CTCF in regulating innate and adaptive immunity. The most common methodology adopted to study the effect of cohesin proteins was the use of conditional deletion of the target protein. This technique involves flanking of the allele with loxP sites that directs deletion to the target cells through the Cre-recombinase system. Immune relevant phenotypes were found for several of cohesin complex and partner proteins (**Table 2**). Apart from lethality, cohesin protein perturbation resulted in reduced innate and adaptive immune cell count, high incidence of lymphomas, increase of spleen size, and improper V(D)J rearrangement. Below, we expand in detail the studies on STAG1, STAG2, RAD21 and SMC3 proteins which are of major interest for development of immune cells and autoimmunity.

**Table 2:** Manipulation of cohesin complex proteins in mouse.

Gene	Manipulation system	Phenotype changes	PMID
*CTCF*	Mox2-Cre controlled recombination system for floxing of *CTCF* in mESC	○ *CTCF^fl/fl^* is embryonically lethal between E4.5 and E5.5 because of increased apoptosis	22532833
CTCF*	Lck-Cre controlled recombination system for floxing of *CTCF* in T cells	○ Thymic T cells of the *Lck-Cre CTCF^fl/fl^* mice are blocked in the immature single positive (ISP) to double positive (DP) transition ○ *CTCF* ^fl/fl^ T cells in ISP stage are reduced in size compared to WT. DP *CTCF* ^fl/fl^ T cells or *CTCF^fl/+^* cells are same size as WT. ○ Low CD3 and TCR expression in *CTCF* ^fl/fl^ DP cells, reduced activation potential ○ Cell cycle defect, not improper *TCR* rearrangement, attributed as the reason for stalling at ISP stage.	18923423
*ESCO2*	Flp recombinase controlled system for floxing of *ESCO2* mice in cerebral cortex and MEF	○ Embryonic lethality of *ESCO2* ^fl/fl^ mice. *ESCO2* ^fl/+^ have no specific phenotype. ○ Weak binding at telomeres and centromeres, enrichment at pericentric heterochromatin of MEF in *ESCO2^fl/fl^*. Chromosome segregation defect ○ ESCO2 mutants reduces SMC3 acetylation and sororin levels in nucleus of MEF	22101327
*HDAC8*	Flp and Cre recombinase controlled system for floxing of *HDAC8* in mESC and NCC	○ *HDAC8^fl/fl^* mice were embryonically viable but die at birth ○ *HDAC8^fl/fl^* in NCC had no differences in phenotype with WT ○ Homeobox TFs were upregulated in HDAC8 mutant skull cell transcripts. ○ HDAC inhibitor treatment results in craniofacial abnormalities in the offspring	19605684
*MAU2*	Flp and Wnt-Cre recombinase system for floxing of *NIPBL* and *MAU2* in mESC and NCC	○ *NIPBL^fl^* ^/fl^ or *MAU2^fl/fl^* mice die at birth. *MAU2^fl/+^* mice show no obvious developmental defects. *NIPBL* ^fl/+^ mice have skeletal malformations.	24700590
NIPBL*	*NIPBL* gene trap insertion into mESC	○ *NIPBL^fl^* ^/+^ mice have no defects in sister chromatid cohesion and DNA repair ○ *NIPBL^fl^* ^/+^ (30% reduction in mRNA and protein level) die frequently at birth. Survivors have heart defects, limb abnormalities, craniofacial defects and have reduced body size, but grow faster after weaning. ○ E14 MEF have among others down-regulated *Ebf1* and *Cebpb* downstream targets IL-6 and *SOCS3*	19763162
*PDS5A*	CMV-Cre controlled system for floxing of *PDS5A* in mESC	○ PDS5B required for centromeric cohesion, and PDS5A for arm and telomeric cohesion. ○ Cell cycle delay due to impaired Aurora B localization in *PDS5B^FL/FL^* cells.	24141881
*PDS5A*	*PDS5A* gene trap insertion into mESC	○ *PDS5A^fl/fl^* or *PDS5B^fl/fl^* embryos have no cohesion defects in MEF, but proper embryonic development requires precise dosage of PDS5 proteins. ○ Important for cardiac and nervous system development	19412548
*PDS5B*	β-actin controlled Cre system to excise floxed *Pds5b* gene in ESC	○ *PDS5B^fl/fl^* is embryonically lethal at E16.5 due to skeletal and cardiac developmental defects. ○ *PDS5B^fl/fl^* mice have no cohesion defects or chromosome abnormality in PDS5B null MEF.	17652350
RAD21*	CD4-controlled Cre recombinase system for RAD21 floxing in thymocytes	○ *RAD21^fl/fl^* DP T cells do not differentiate into SP stages. The cells are nondividing, die after anti-CD3 stimulation. ○ *RAD21^fl/fl^* T cells have affected *Tcra* rearrangement and transcription	21832993
*RAD21*	TEV protease mediated cleavage of RAD21/Scc1	○ *RAD21^-/-^* mice embryos were viable and have no defects of meiotic chromosome segregation. ○ Sister chromatid segregation affected after RAD21/Scc1 cleavage.	20971813
*SGO1*	Gene trap insertion to truncate *Sgo1* expression in MEF.	○ Embryonic lethality of *SGO1 ^fl/fl^* mice, *SGO1^fl/+^* mice were normal. ○ Mild chromosome segregation defects in *SGO1^fl/+^* MEF. ○ *SGO1^fl/+^* mice had higher frequency of colonic tumors. ○ *SGO1^fl/+^* mice show higher IL6, p53, Bcl2	22262168
*SGO2A*	Gene trap insertion to truncate *Sgol2* expression in mESC	○ *SGOL2^-/-^* were sterile, while *SGOL2^+/-^* were fertile. ○ *SGOL2^-/-^* mice have normal mitosis but have meiotic defects.	18765791
*SMC1A*	No manipulation.	○ ChIPseq peaks for SMC1A, CTCF, H3K27me3, H3K27me2, and H3K27ac from mouse embryonic limbs were overlapped and analysed. ○ Cohesin involved in distal sites interacting with promoters across different tissues. ○ *Pcdh* gene cluster, *Wnt7a*, *Snai1* are some examples where multiple tissues show similar chromatin interaction profiles.	23704192
*SMC1B*	Spontaneous recessive mutation in *SMC1B* observed in a mouse colony was analysed.	○ SMC1B^-/-^ and SMC1B^+/-^ mice are sterile. ○ Embryonic death was not observed.	19491376
*SMC1B*	Vector designed to target and excise exon 10 of *SMC1B* in mESC	○ SMC1B*^-l-^* are sterile, while SMC1B^+/-^ are fertile. ○ Embryonic death was not observed. ○ SMC1B is dispensable for establishing sister chromatid cohesion but required for cohesion maintenance. ○ In SMC1B^-/-^, early cell cycle was normal, but cell cycle progression stops at pachytene stage. while other cohesin protein localization was not affected	15146193
SMC3*	CD19 or Cγ controlled Cre recombinase system for floxing of *SMC3* and isolation of bone marrow/spleen cells.	○ *SMC3^fl/fl^* is embryonically lethal, and *SMC3^fl/+^* have abnormal craniofacial morphologies. ○ Spleen morphology is unchanged in *SMC3^fl/fl^* and *SMC3^fl/+^* mice. *SMC3^fl/fl^* mice lack GC formation. *SMC3^fl/+^* mice have high proliferative GCs and no apoptosis. ○ Differentiation into plasma cells was affected, plasmablasts accumulated in *SMC3^fl/+^* mice, but not affinity maturation or class switch recombination. ○ Lineage TF Pax5 was decreased in plasmablasts of heterozygous vs WT, and differentiation-specific TF Irf4, Prdm1 were increased.	33432228
*SMC3*	Analysis of mutant mouse lines (Mouse Genome Project)	○ Mutations in human may not be as lethal as similar mutations in mice, but some phenotypes are similar, and some are extra in mice. e.g., increased CD4 and CD8 counts seen in *Smc3* mutant heterozygotes	23870131
STAG1*	Gene trap insertion to excise *STAG1* in mESC siRNA knockdown of STAG1 or STAG2 in MEFs	○ Cohesin-STAG1 important for telomeric cohesion, cohesin-STAG2 important for centromeric cohesion. STAG1 and STAG2 have non-redundant chromatin binding sites ○ *STAG1^fl/fl^* mice are embryonically lethal at E12.5. Centromeric cohesion defects not seen, but chromosome segregation affected. ○ *STAG1^fl/fl^* MEFs have downregulated Myc target genes. ○ IL-6 upregulated in STAG1^-/-^ MEFs ○ Transient knockdown of either STAG1 or STAG2 causes no expression changes	22415368
*STAG1*	Gene trap insertion to excise *STAG1* in mESC siRNA knockdown of STAG1 or STAG2 in MEFs	○ *STAG1^fl/fl^* MEFs have reduced proliferation. *STAG1^fl/+^* mice have early hematological malignancies ○ Telomere structure and replication defects in STAG1^-/-^ MEF.	22415365
STAG2*	Mx1-Cre controlled recombination system for floxing of *STAG2* in MEF	○ Chromosome segregation not affected, but minor fraction shows mild cohesion defects. No early tumor onset. ○ Trilineage hematopoiesis and extramedullary erythropoiesis is affected. ○ Proliferative cells in bone marrow, lymph nodes, spleen are affected. ○ T cells count decrease in peripheral blood and spleen; monocytes and neutrophils increase ○ *STAG1* deletion has no organ malformation, but *STAG2* deletion has widespread heart defects, and lethality at E9.5.	32783938
STAG2*	Mx1-Cre controlled recombination system for floxing of *STAG2*.	○ Stag2^fl/fl^ mice have low WBC, low hemoglobin, enlarged spleen, survival not affected. ○ Myeloid skewing with dominance of monocytes and neutrophils. ○ STAG2 highly enriched at high K27ac sites in meaning active enhancers/promoters. STAG2 and STAG1 equal at high CTCF sites. ○ IRF motif enrichment in ATACseq, *Hox* cluster downregulation. ○ Inflammatory response and IFN response are altered in STAG2^fl/fl^ mice.	32249213
*STAG2* *SMC1A* RAD21*	shRNA vector for SMC1A, RAD21, STAG2 (75-90% suppression in HSPC)	○ shRNA of both SMC1A and STAG2 caused enlarged spleen, myeloid hyperplasia, lymphopenia. ○ Differentiation into erythroid and lymphoid lineages affected. Self-renewal increased. ○ No chromosomal instability ○ Altered GATA1 motif accessibility after shRNA-STAG2 in HSPC. ○ Splenic morphology, bone marrow hyperplasia seen in shRNA-SMC1A mice. Myeloid lineage cells found in high numbers.	26438359
STAG1 AND STAG2*	Mx1-Cre controlled recombination system for floxing of *STAG1* or *STAG2*	○ Stag1^fl/fl^ has no hematological specificity. Stag2^fl/fl^ has HSC expansion, bone marrow failure, Increased self-renewal and reduced differentiation. ○ B-cell lineage commitment genes (Pax5, CD19 etc.), and myeloid and erythroid lineage (*Ccr2*, *Fcgr2b*, *Hbb-b1* etc.) commitment genes were decreased in Stag2^fl/fl^ mice. ○ Genome sites that uniquely bind STAG2 (and not STAG1), have altered local chromosomal interactions after STAG2 KO, which could lead to altered transcription through CTCF-independent factors, for example PU.1 and Ebf1.	31495782
*WAPL*	Estrogen Receptor-Cre controlled recombination system for floxing of *WAPL* in mESC	○ *WAPL^fl/fl^* is embryonically lethal. ○ *WAPL^fl/fl^* causes formation of vermicelli structures because of change in cohesion location. ○ Increased formation of cohesin-mediated loops, chromatin compaction and affects sister chromatid separation. ○ cMyc reduced in *WAPL^fl^* ^/fl^ MEF. Ectopic supplementation of WAPL promotes cell cycle progression.	23975099

*These studies are described in the text.

mESC, mouse embryonic stem cells

MEF, mouse embryonic fibroblasts.

Complete deletion of either *Stag1* or *Stag2* leads to prenatal mortality of mice. Thus, studies on mouse embryonic fibroblasts (MEF) indicated that STAG1 was likely involved in cohesion of telomeres, while STAG2 accounts for centromeric cohesion (61). Cre-lox mediated deletion in MEF at E12.5 revealed non-redundant differences in distribution of chromatin binding between STAG1 and STAG2. However, cohesin-perturbed transcriptomes in general do not show widespread alterations (62), with tissue specificity and altered chromatin interactions being more important factors. For example, siRNA-induced acute deficiency in STAG1 caused significant changes in mRNA levels of 15 genes to known immune mediators including *Mmp3*, *Ccl6*, *Itgam*, *Sox11*, *Bcl2a1b*, and *Fcer1g* (63). Additionally, STAG1 widely bound transcription factor (TF) Myc in ChIP experiments, and concomitant Myc expression and binding was abolished in STAG1-null MEFs (63). These Myc-dependent genes are essential in IFN signaling and inflammatory response. Studies on mouse haematopoietic stem cells of *Stag1*-null mice showed that these genes require the presence of key TF RUNX1, and the formation of proper enhancer-promoter loops (51). In contrast, only 2 genes (*Casp1* and *Ctss*) showed significant transcriptional changes after siRNA-induced depletion of *Stag2* in MEFs (63). *Stag2* deletion in adult mice (4 weeks) by tamoxifen-Cre system affected bone marrow haematopoiesis and highly proliferating cells of spleen and lymph nodes combined with rapid decrease in content of T cells, while monocytes and neutrophils increased (64). *Stag2* deletion in haematopoietic stem and progenitor cells (HSPC) using Mx1-Cre system resulted in a complete loss of trilineage haematopoiesis in the bone marrow, suppression of lympho-, thrombocyto- and erythropoiesis and overwhelming predominance of myeloid cells controlled by RUNX1 and GATA2 (51). Similarly, HSPC treated with shRNA for STAG2 (65) displayed altered chromatin accessibility and reduced expression of related genes important for myeloid differentiation including *Fcgr3/4*, *Mpo* and *Gata1*. STAG1 and STAG2 are functionally non-redundant. Absence of STAG1 causes STAG2 to accumulate at intergenic regions but does not substitute for function which results in an increased disruptive effect causing both myeloid pathology and solid cancers (66). Eliminating STAG2 causes embryonic lethality and proliferative defects in mouse embryonic fibroblasts *in vitro* acting through disruption of chromatin accessibility for REG and IRF8 transcription factors (64) and devastating consequences for lineage-dependent hematopoietic gene expression programs (30). This effect was unique to STAG2, as STAG1 retained maintenance of chromatin interaction boundaries even under absence of STAG2. Taken together, these studies highlight the need for investigating core cohesin complex proteins and/or its associated partners within the plethora of disease-relevant haematopoietic cell types.

The role of RAD21 in a myeloid context was analysed in a recent study where mature nonproliferating macrophages and HSPC of RAD21-null or heterozygote mice were examined using multiomic techniques (49). *Rad21* deletion caused deregulation in a broad panel of inducible genes controlling inflammatory responses by changing chromatin accessibility in crucial enhancer regions containing motifs for ISRE, STAT, IRF and PU.1. Subsequent TLR4 activation in *Rad21*-null macrophages with LPS showed reduced expression of *Irf7*, *Stat1 and Stat2*. Large scale chromatin contacts remained comparable after activation of *Rad21*-null and WT macrophages, while local chromatin interactions were affected, specifically at immune-relevant loci containing *Cebpb* and *Egr2* genes. *Rad21^+/-^* mice showed reduced expression of inflammatory cytokines IL-10 and IL6, indicating possible dosage effects. At the early developmental immune stage of HSPCs, acute inhibition of RAD21 decreased expression of central differentiation TFs *Prdm1, Fos, Jun*, and important immune mediators *Myc* and *Irf2* and *Ifngr1.* In a lymphoid context, where *Rad21* deleted in thymocytes under a CD4 regulatory element controlled Cre recombinase expression system (67), progression of thymocytes from the double positive (DP) to the single positive (SP) stage was ablated. The deletion also caused chromatin rearrangement in the *Tcra* gene segments with subsequent impact on transcription. Thus, proper amounts of RAD21 is required in haematopoietic cell types to mediate effective distant chromatin interactions and ensure successful differentiation into mature cell subsets.

Similar to *Rad21*-null thymocytes (67), conditional deletion of *Ctcf* in T cells impaired the progression of T cells from the immature single positive to the DP stage (68). Additionally, *Ctcf*-deficient thymocytes exhibited a reduced size phenotype compared to heterozygous *Ctcf* ^fl/+^ or WT cells. Homozygote deletion of *Ctcf* caused reduced CD3 and T Cell Receptor (TCR) expression, with consequent reduction in activation potential. Defects in cell cycle, rather than improper *Tcra* rearrangement, were attributed as causes for impaired T cell development.

*Smc*3 deletion in the bone marrow B cells through CD19-Cre system of adult mice displayed an intriguing immunologically relevant phenotype (31). Homozygous absence of SMC3 completely abolished immunization-induced germinal centre formation. Heterozygous mice, in contrast, had highly proliferative germinal centre cells and reduced differentiation into plasma cells. Interestingly, apoptosis was not observed, indicating that reduced SMC3 levels were still tolerable for cell survival. However, the crucial mechanisms of B cell affinity maturation and class switch recombination was not SMC3 dependent. B cell lineage specific TFs EBF1 and Pax5 were decreased in heterozygous cells compared to WT while differentiation-specific TF IRF4 and PRDM1 were increased, pointing on the role of SMC3 levels in developmental progression of B cells.

Effects of dysfunctional NIPBL obtained through truncated *Nipbl* transcripts, replicated prominent structural and functional abnormalities observed in CdLS patients (34). Unlike other cohesin proteins where heterozygosity was largely tolerated, heterozygous *Nipbl* mice died frequently at birth while survivors displayed cardiac and craniofacial defects. Growth rate defects observed at birth disappeared after weaning. Among the genes affected in *Nipbl* MEF were downregulated *Ebf1, Cebpb, Pparg, Il6* and *Socs3*, which play important roles in glucose metabolism and adipocyte differentiation. Concordantly, *Nipbl* MEFs had poor spontaneous adipogenesis *in vitro*. These genes have been widely explored in inflammation and autoimmune processes triggering development of diabetes mellitus and RA.

## Immune Loci Regulation by the Cohesin Complex

Differentiation of HSPCs is important in generating the erythroid, lymphoid, and myeloid cell subsets important for innate and adaptive immunity. Cohesin-CTCF plays a central role in organizing and executing gene expression programs in each of these processes. For example, differentiation into erythroid cells from HSPC requires intact functional cohesin complex proteins and related co-organizing TFs such as KLF1 and GATA1 (69). Converging data from cells of patients with different myeloid syndromes, CdLS patients, lymphocytic cell lines and from cells from immune tissues of mice indicate that cohesin complex subunits and associated protein factors play crucial roles in organizing genome three-dimensionally, in facilitating enhancer-promoter interactions, and in expression of immune-relevant genes.

Below, we present classical examples of immune loci regulated by cohesin-CTCF complex.

### Antigen Receptor Loci

**Figure 2:**
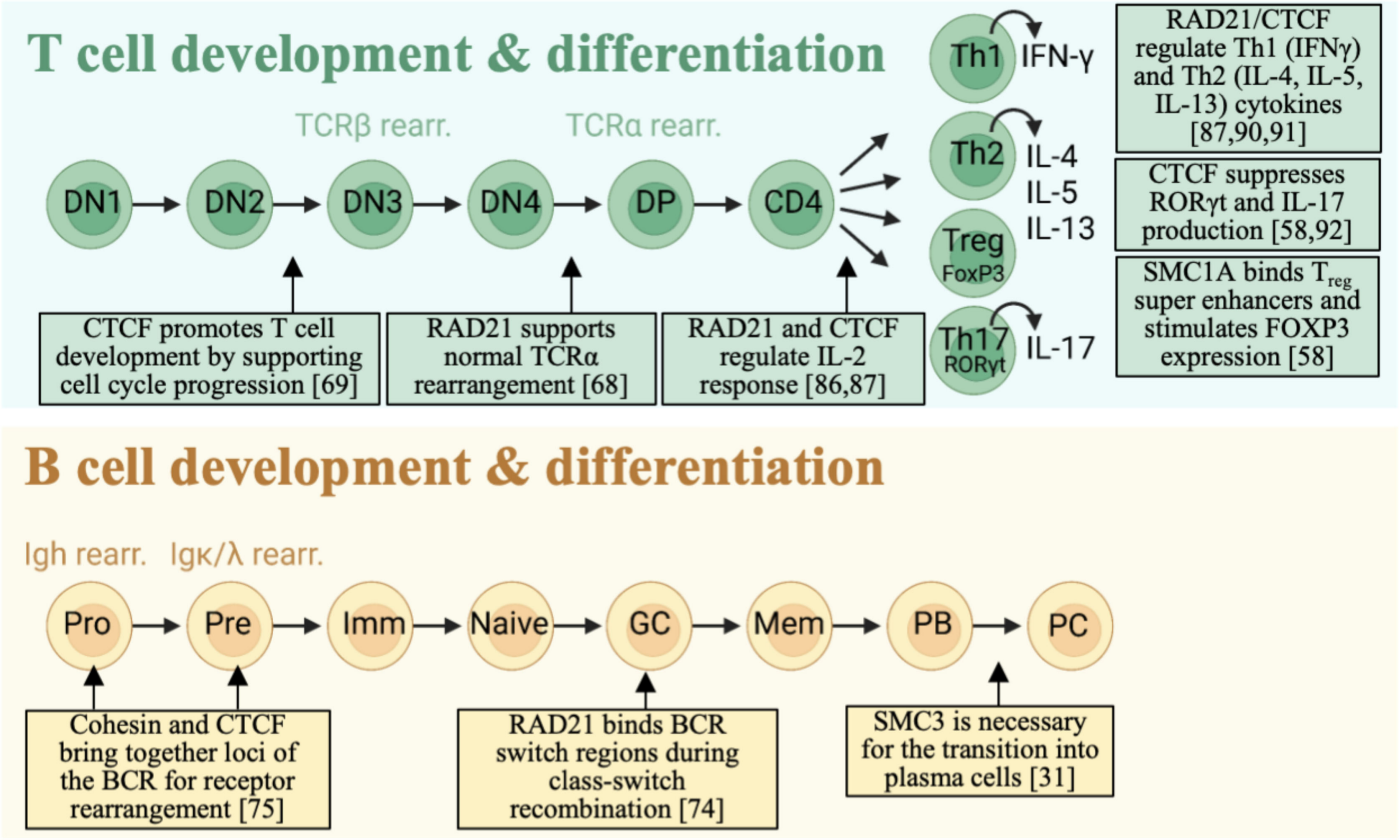
Cohesin complex involvement in T and B cell development. Cohesin-CTCF complex is important for successful transition through lymphocyte developmental stages. RAD21 and CTCF ensure proper TCR rearrangement by organizing distant V and J segments into close interacting networks. In B cells, RAD21 and SMC3 bind to specific BCR loci and regulate differentiation.

The TCR binds to antigens/peptides presented by the major histocompatibility complex (MHC) with high specificity. It is composed of an α (TCRα) and β (TCRβ) chain that both have a constant and a variable region. The high variability of the TCR that allow the T cell population to recognize a vast number of pathogens is achieved by somatic recombination (rearrangement) of variable (V), diversity (D) and joining (J) segments of the TCR gene. Rearrangement of the *TCR* gene is aided by RAG recombinases and occurs during T cell development in the thymus (**Figure 2**). Circulating lymphoid progenitors enter the thymus and adopt a developmental pathway, which is characterized by acquisition of the major cell surface markers CD4 and CD8. Rearrangement of the TCRβ chain occurs during the double negative (DN, CD4^-^CD8^-^) phase following the entry to the thymus. Rearrangement of TCRα chain occurs during transition of the immature single positive (ISP) T cell into the double positive (DP, CD4^+^CD8^+^) phase. Following these events, the T cell undergo positive selection for TCR carrying cells. At this point, the T cell becomes single positive for either CD4^+^ or CD8^+^, migrate into the medulla of the thymus and undergo negative selection for T cells that are reactive to the host. After passing the negative selection stage, mature T cells migrate to secondary lymphoid organs.

The role of cohesin in TCRα rearrangement was studied by a conditional knockout (KO) of *Rad21* that was activated by the expression of CD4 when the T cells transition from the DN to DP phase (67). RAD21 depletion during TCRα rearrangement skewed the selection of the J segment in TCRα toward more distal segments and reduced H3K4me3 methylation and consequently reduced recruitment of RAG recombinases. Furthermore, the cells typically did not survive cell division occurring in the SP phase. Mice with a conditional KO of CTCF in thymocytes at all developmental stages with full deletion accomplished at the DN2 stage have reduced proportions of cells entering the DP and SP stages. This block was seemingly independent of TCR rearrangement but rather depended on cell-cycle arrest (68). Cell-type specific gene expression is the key to proper development of T cells. It has been shown that expression of the *Rag* gene relies on binding of the transcription factor E2A to the enhancer-promoters junction. The enhancer-promoter interaction at that site is directed by the cohesin-CTCF complex and acquires properties of super-enhancers (70). Proper rearrangement of the TCR chains is essential for generating a diverse TCR repertoire which is an important step of the earliest thymocyte development. This developmental checkpoint establishes the fate of T cells as αβ or γδ and contributes critically towards autoimmunity (71, 72). A broad understanding of functions for cohesin complex proteins in a T-cell specific and TCR-specific manner is still lacking.

The B cell receptor (BCR) exists in two isotypes, immunoglobulin IgM or IgD, and consists of two light (Ig kappa or Ig lambda) and two heavy (IgH) Ig chains that are dependent on their association to a heterodimer of Igα/Igβ for intracellular signal transduction (73) (**Figure 2**). Upon cognate antigen binding, conformational changes cause activation of the BCR, with immunoreceptor linked tyrosine motifs (ITAMs) present on Igα/Igβ initiating a cascade of immune signaling pathways (73). Similar to the TCR gene rearrangement described above, developing B cells in the bone marrow perform sequential rearrangement of their Ig heavy and light chain loci, commonly termed V(D)J recombination (25), which is essential to the generation of antibody diversity. The pro-B stage is characterized by heavy chain re-arrangement, and the subsequent pre-B stage is characterized by the rearrangement of the light chain (kappa or lambda). B cell activation that occurs after the B cell has migrated from the bone marrow to secondary lymphoid organs will lead to further editing of the BCR during which the BCR switch isotype (class) and undergo somatic hypermutation of the variable region, these are essential steps as the B cell progress toward becoming an antibody-producing plasma cell.

Cohesin and CTCF has been suggested to play an important role in antigen receptor loci rearrangement because genes spanning large regions of the genome needs to be brought into proximity for the rearrangement to take place. Indeed, ChIP-chip experiments revealed high density of CTCF bound across the light and heavy Ig loci, although binding patterns were similar at different developmental stages. Interestingly, RAD21 and CTCF co-localized during the pro-B cell phase during which the heavy chain rearranges, but little RAD21 binding was found in CTCF binding sites in the pre-B phase, indicating a stage specific role for cohesin in BCR rearrangement (74). Specifically, colocalization was largely observed within the variable (V) regions of the heavy chain during the pro-B phase, and inhibition of CTCF by shRNA led to longer distances within the variable locus of the heavy chain supporting the idea that cohesin CTCF play an important role in BCR rearrangement (75). The light kappa chain locus also showed stage specific binding of CTCF/cohesin, with most binding during the pre-B cell stage during which the light chain rearranges (75). It was observed that reverting to a previous cellular developmental state does not concomitantly result in adoption of the earlier genomic reorganization that was altered by loss of CTCF-cohesin interaction during development (25). This discrepancy could be explained by the impaired expression of lineage-determining TFs GATA3, TCF1/LEF1, Ikaros etc, which require the initial chromatin architecture established by cohesin-CTCF. In another study, it was shown that altered CTCF binding sites cause disruption of the interaction between V_H_ and D-J_H_ loci at the 890kb *Igh* region and contribute to the propagation of immature B cells (76).

Splenic B cells activated for 48h with IL-4 and LPS to promote class-switch to IgG1 lost the enrichment of RAD21 binding in promoter regions as observed in unstimulated B cells. Specifically, binding of RAD21 was underrepresented in switch regions and in association with PRDM1 and PAX5, both involved in the transition to plasma cells (73). A different study used a conditional KO of SMC3 that were activated by the expression of Cγ1 in B cells during the germinal centre (GC) reaction (31). While *Smc*3^-/-^ mice completely lost the GC formation, *Smc*3+/- mice had larger GCs caused by increased proliferation, but impaired transition into plasma cells possibly due to a failure of inducing *Prdm1*.

### Major Histocompatibility Complex, MHC

The MHC gene cluster is divided into three classes of genes of which the first two are involved in antigen presentation to T cells. MHC class I (MHC-I) molecules are expressed in all nucleated cells and present peptides resulting from proteasome degradation (antigens) to alert CD8^+^ T cells of infection or malignancies. MHC class II (MHC-II) molecules are expressed mainly by professional antigen-presenting cells and present peptides to CD4^+^ T cells. Polymorphism in the genes involved in antigen presentation contribute to a high diversity of the biding affinity to peptide antigens. In human, MHC is referred to as human leukocyte antigen (HLA) and the *MHC-I* genes code for the highly polymorphic classical HLA genes *HLA-A*, *-B*, *-C*, and the non-classical *HLA-E*, *-F* and *-G* with limited polymorphism. The *MHC-II* genes include the polymorphic classical HLA genes *HLA-DR*, *-DP* and *-DQ* (77). The high level of polymorphism of MHC genes is associated to various autoimmune disorders. For example, polymorphism in *HLA-DRB1* is associated with increased risk for RA (78), SLE (79), T1D (80) and multiple sclerosis (MS) (81).

The core cohesin proteins RAD21 and SMC3 have been identified localized at all *MHC-II* insulator segments in immortalized mouse B cells (82). Cohesin presence at these insulator regions requires the co-binding of CTCF. Importantly, siRNA-mediated reduction of RAD21 and SMC3 levels weaken the chromatin loops formed between the promoter proximal gene sequences of *HLA-DRA* and one of the MHC-II insulators. This, in turn, affects *MHC-II* gene expression. As potential CTCF-mediated interaction between insulators in MHC-II locus was observed to be unchanged in the absence of RAD21, the findings emphasize the need for the stability of chromatin loops rendered by cohesin complex binding to obtain efficient gene expression.

### IL-2

IL-2 promotes the differentiation of naïve T cells into effector cells following TCR activation, and may, in combination with additional cytokines, induce the polarization into the Th1 and Th2 phenotypes of CD4 cells. It plays an important role in maintaining self-tolerance by inducing the formation of regulatory T cells in the thymus and limiting the production of pro-inflammatory cytokines by Th17 cells. Furthermore, IL-2 has an important role in regulating metabolic programs of T cells (83).

Activated and differentiated T-helper cells possessed differentially expressed accessible chromatin region at enhancers and introns compared to quiescent counterparts (84). This indicates a rapid remodelling of the genome under stimulation. NF-kB signaling and IL-2 cytokine family are a few of the identified enriched pathways after stimulation. This study corroborates the observation that when Th1 cells are supplemented with a high concentration of IL-2, CTCF and RAD21 binding is essential for IL2-sensitive gene transcription. *In vitro* siRNA-induced inhibition of RAD21 or CTCF in primary mouse spleen CD4^+^ and CD8^+^ T cells affected sensitivity to IL-2 stimulation and resulted in reduced expression of the IL-2 sensitive genes. These results indicate important regulatory role of cohesin complex in mediating IL2 signaling (85). Furthermore, CTCF-deficient deletion in mature CD4^+^ and CD8^+^ T cells had a reduced expression of the IL-2 receptor CD25 following TCR activation but expressed normal levels of IL-2 (86).

### Th1 and Th2

Single positive naïve CD4^+^ T cells possess the plasticity to interconvert between the several T-helper subsets distinguished by expression of key transcription factors and pattern of cytokine production. Th1 cells express the master transcription factor T-bet while Gata-3 acts as the master transcription factor for Th2 cells. This cytokine-determined Th1 and Th2 phenotype of T cells define their roles and functions in autoimmune pathology (87, 88)

Interferon-γ (IFNγ) production is characteristic for Th1 cells. Two studies described presence of cohesin/CTCF binding sites in proximity of *IFNγ* and demonstrated their importance for regulation of IFNγ production (89, 90). ChIPseq and RT-PCR analysis of the human non-lymphoid 293T cell line and primary CD4^+^ T cells show strong binding of RAD21 and CTCF across the *IFNγ* region, with significantly more abundance in Th1 cells, which could explain high expression levels of IFNγ by these cells (90). These sites were also occupied by the Th1 specific TF T-bet (89). Studies with 3C capture assay demonstrated interaction between these CTCF/cohesin sites in proximity of the *IFNγ* that was reduced in Th2 cells, the subtype not producing IFNγ. The interaction was dependent of CTCF-expression, and deletion of CTCF decreased the IFNγ expression. Taken together, these studies demonstrated presence of Th1 specific chromatin looping at the *IFNγ* locus necessary for IFNγ production by CD4^+^ T cells.

Th2 cells are characterized by production of IL-4, IL-13 and IL-5, whose encoding genes are located in cluster on chromosome 5. ChIPseq studies revealed the presence of multiple CTCF binding sites in the Th2 cytokine locus. Conditional knockout of CTCF in DP thymocytes had significantly augmented ability to differentiate into IL-4, IL-5, IL-13 producing Th2 cells or into IFNγ producing Th1 cells (86). In a different study, expression of IL-17 was enhanced by CTCF depletion (91). Regulation of IFNγ and IL-17 production could be explained by the requirement of DNAseI hypersensitive region containing the binding sites for CTCF and assisted by Oct1/PUO2F1 mediated inter-chromosomal interaction with the Th2 cytokine locus.

### T_reg_ and FOXP3

FOXP3 is called the master transcriptional regulator of T regulatory cells (T_regs_), which implies its major contribution to differentiation of CD4^+^ T cells into T regulatory cells. Mutations or distortions of the *Foxp3* locus in man and mouse leads to a multiple autoimmune disorder clinically presented as diarrhoea, diabetes, and eczema (92).

Regulation of *FoxP3* is not completely understood. It was observed that FoxP3-deficient T cells possessed a similar regulatory chromatin landscape to their sufficient controls (93). FOXP3 itself has minor effects on the 3D genome organization and therefore depends on other TFs and CTCF for chromatin accessibility. CTCF establishes and maintains open chromatin regions in nonlymphoid tissue Tregs (94), since the bulk of FOXP3 binding sites are found in repressed/poised chromatin (93). CTCF overexpression in CD4^+^ T cells increased expression of Foxp3 and decrease Rorγt (57). CTCF binding sites colocalized together with T_reg_-specific super-enhancers were required for expression of the T_reg_-signature genes including *Foxp3*, *Ctla4*, *Il2RA* and *Ikzf2*, suggesting a reliance on cohesin/CTCF complexes for chromatin looping to facilitate proximity between super-enhancers and promoters (95).

## Protein-Protein Interaction Network of the Cohesin Protein Complex

Selective studies after manipulation of cohesin complex proteins has provided valuable insights into the role of cohesin in genome architecture. However, it has also masked the relevance of a large-scale exploration of the many other proteins that could potentially interact with the cohesin complex. Additionally, there exists a lack of human studies on the role of cohesin complex proteins in immune system development and immune response regulation, with only sporadic information extracted from mouse models being targeted to immune competent cell populations. This urged us to analyse potential functional importance of the core cohesin proteins for immunity through construction of protein-protein interaction network, considering an extended list of cohesin-related proteins and their protein interacting partners.

**Figure 3:**
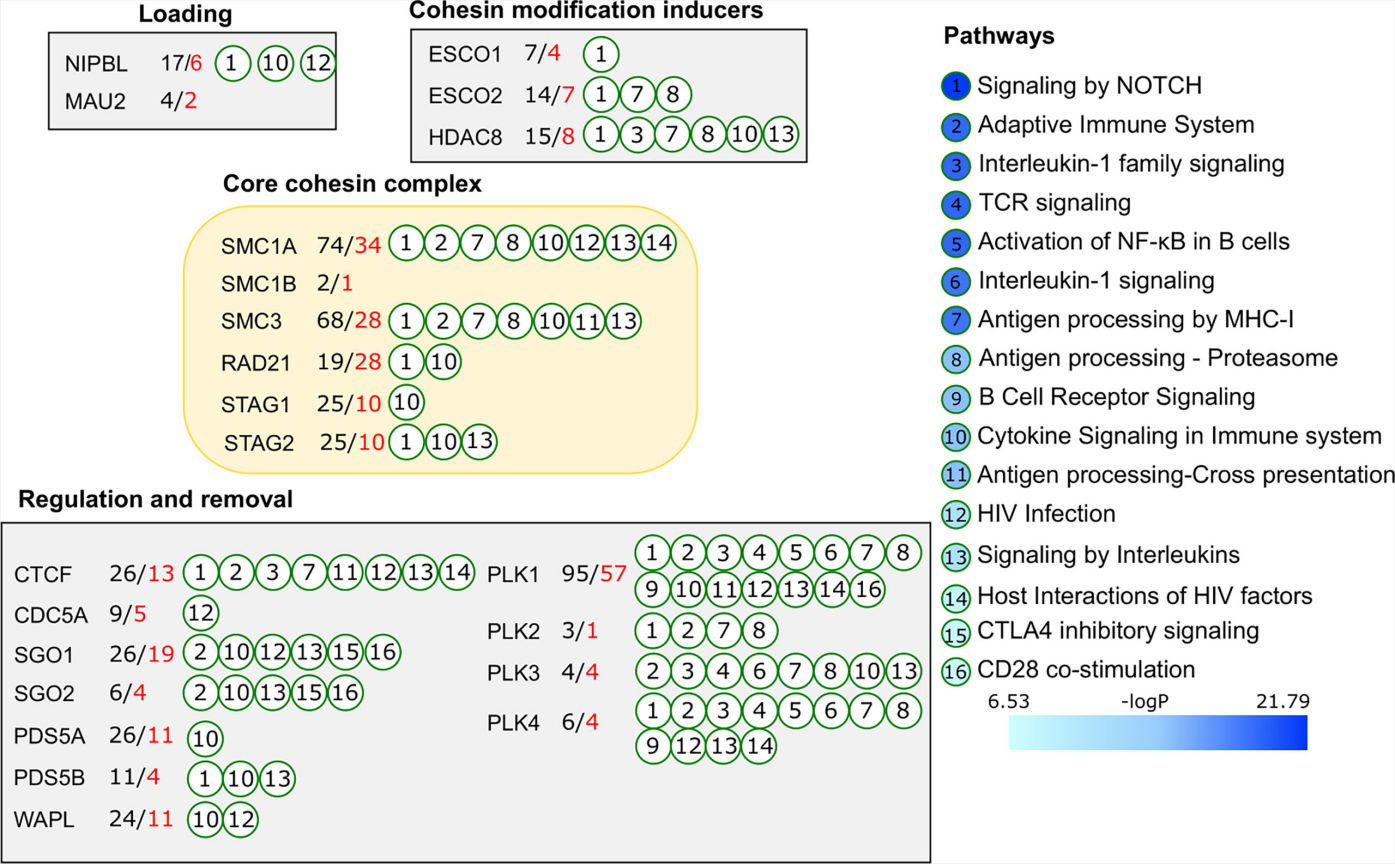
Functional enrichment analysis of the cohesin complex proteins. Experimentally confirmed protein-protein interactions for 22 proteins of the cohesin complex were retrieved from the BioGrid database. Functional enrichment analysis was done in Reactome. Immunologically relevant pathways (FDR p-value <10^-5^) are presented on the right side and annotated to individual cohesin complex proteins by circled numbers corresponding to the immunologically relevant pathways. Number of interactors is shown next to each protein. Total number of interactors is indicated in black, immunologically relevant interactors are shown in red numbers.

To avoid noise and to reduce non-specific binders, we extracted only the interactions supported by two or more experimental evidence in the BioGRID database (**Figure 3**). Starting from the 22 proteins of the cohesin complex, we created a list of 246 confident cohesin binding interactors. In addition to obvious interactions within the cohesin complex, the list presented connections with other biological processes engaging cohesin proteins. Some were involved in multiple interactions, e.g., 74 for SMC1A, and 68 for SMC3 while others had fewer number of interactors (**Figure 3**). Loading complex proteins MAU2 and NIPBL were associated with 4 and 17 interactors, respectively. Among factors regulating cohesin binding and removal, PLK1 was characterized by 95 interactors in contrast to other members of the PLK family, which had between 3 and 6 interactions each. Indeed, PLK1 is known to be involved in multiple non-cohesin functions and it appeared here as one of the key proteins connecting cohesin with immune relevant processes. Several cohesin proteins had common interactors among which were SF3B1 and SF3B3 slicing factors, dead box helicase 47, and nuclear matrix SRRM1. Notably, autoimmune regulator AIRE was identified as a common interactor of SMC1A, SMC3 and RAD21, and was also functionally validated in mouse thymic epithelial cells (96). These observations allowed us to anticipate that the list of common interactors will enhance sensitivity for functional analysis.

Based on the extended list of cohesin complex proteins and their interactors, we performed functional enrichment analysis in MetaScape service (97) with the total human proteome in background. Gene Ontology and pathway databases KEGG, Reactome, and Wiki Pathways were selected for the analysis of functional categories. Expectedly, the top enriched categories included cell cycle, mitosis, chromosome segregation and organization groups (Reactome Cell Cycle: logp= -91.7, GO : BP sister chromatid segregation: logp= -49.15). However, immune system and related functional categories were also represented in the top group and included, among others, Adaptive Immune System, Interleukin-1 family signaling, TCR signaling, and Signaling by Interleukins (**Figure 3**). In total, 137 of 246 (55.7%) cohesin binding interactors were involved in the immune processes significantly enriched among functional categories. CDCA5, PLK1, SGO1, SMC1A and STAG2 were the core cohesin proteins associated with antigen processing by MHC, cytokine signaling, adaptive immunity, and others, mediated through hub cohesin proteins and their interaction partners.

Considering the focus of the described approach on strict filtering for high confident protein interactors, this result highlights a tight association between the cohesin complex and immune system regulation and presents a strong support to the cohesin-centric mouse studies described above.

## Conclusions

In this review, we explored and summarized current knowledge connecting the proteins of cohesin complex with immune relevant processes and transcriptional activity in immune loci which has just started emerging. Functional *in vivo* studies on the proteins of the cohesion complex are complicated by embryonic lethality, thus most of the information today is collected through work on embryonic and progenitor cells. Inhibition of individual cohesion proteins in mice revealed important physiological role of these proteins in early stages of haematopoiesis (STAG2, RAD21, SMC1, SMC3), adipogenesis (NIPBL) and inflammation (RAD21). In this context, cohesin proteins act at the intersection of major immune pathways including those mediated by NF-κB (RAD21), IL-6 (STAG1, SGO1, RAD21) and IFNγ (RAD21, STAG2). Additionally, cohesin proteins participate in thymocyte maturation (CTCF, RAD21, SMC3) and B cell (SMC3) development, signifying their requirement for stage maturation of the immune cells. Human GWAS is remarkably concordant with these observations and identify associations between SNPs in the genes coding for cohesin with leukocyte traits, C-reactive protein and canonical autoimmune conditions as RA, MS, asthma and HLA-B27 associated inflammatory conditions. Considering evolving advanced techniques to investigate the highly ordered chromatin environment, we have presented ample evidence supporting the notion that proteins of the cohesin complex will present new clues in regulation of adaptive immune processes and offer novel therapeutic strategies to prevent and cure autoimmunity.

## Author Contributions

MB and GK conceived the manuscript. MB, VC, NO, CW, EMB, KMA did literature research and wrote the first draft. M-JG-B, CW, VC, and MCE prepared the figures. NO, VC, and MB drafted and edited tables. All the authors were involved in discussions and contributed to the revision of the manuscript.

## Funding

This work has been funded by grants from the Swedish Research Council (MB, 2017-03025; 2017-00359; CW, 2020-00592), the Swedish Association against Rheumatism (MB, R-860371), the King Gustaf V:s 80-year Foundation (MB, FAI-2020-0653), and the Regional agreement on medical training and clinical research between the Western Götaland county council and the University of Gothenburg (MB, ALFGBG-717681). 

## Conflict of Interest

The authors declare that the research was conducted in the absence of any commercial or financial relationships that could be construed as a potential conflict of interest.

## Publisher’s Note

All claims expressed in this article are solely those of the authors and do not necessarily represent those of their affiliated organizations, or those of the publisher, the editors and the reviewers. Any product that may be evaluated in this article, or claim that may be made by its manufacturer, is not guaranteed or endorsed by the publisher.
